# The realistic performance achievable with mycobacterial automated culture systems in high and low prevalence settings

**DOI:** 10.1186/1471-2334-10-93

**Published:** 2010-04-12

**Authors:** Sanne C van Kampen, Richard M Anthony, Paul R Klatser

**Affiliations:** 1 VU University Amsterdam, Faculty of Earth and Life Sciences, De Boelenlaan 1085, 1081 HV Amsterdam, The Netherlands; 2 Royal Tropical Institute (KIT), KIT Biomedical Research, Meibergdreef 39, 1105AZ Amsterdam, The Netherlands

## Abstract

**Background:**

Diagnostic tests are generally used in situations with similar pre-test probability of disease to where they were developed. When these tests are applied in situations with very different pre-test probabilities of disease, it is informative to model the likely implications of known characteristics of test performance in the new situation. This is the case for automated *Mycobacterium tuberculosis *(MTB) liquid culture systems for tuberculosis case detection which were developed and are widely used in low burden settings but are only beginning to be applied on a large scale in high burden settings.

**Methods:**

Here we model the performance of MTB liquid culture systems in high and low tuberculosis (TB) prevalence settings using detailed published data concentrating on the likely frequency of cross-contamination events.

**Results:**

Our model predicts that as the TB prevalence in the suspect population increases there is an exponential increase in the risk of MTB cross-contamination events expected in otherwise negative samples, even with equivalent technical performance of the laboratories. Quality control and strict cross-contamination measures become increasingly critical as the burden of MTB infection among TB suspects increases. Even under optimal conditions the realistically achievable specificity of these systems in high burden settings will likely be significantly below that obtained in low TB burden laboratories.

**Conclusions:**

Liquid culture systems can play a valuable role in TB case detection in laboratories in high burden settings, but laboratory workers, policy makers and clinicians should be aware of the increased risks, independent of laboratory proficiency, of cross-contamination events in high burden settings.

## Background

Automated liquid culture for *M. tuberculosis *(MTB) is widely used in industrialized countries and increases the sensitivity and reduces the time required for culture based tuberculosis (TB) diagnosis. The use of this highly sensitive technique increases the importance of robust protocols and quality control to prevent and if this occurs detect laboratory cross-contamination. Protocols and quality control systems have been developed over the past years that are adequate to ensure excellent performance in settings with a low burden of TB. With the recent trends in TB diagnostics for high burden countries moving towards more modern technologies and scale-up of laboratories for the implementation of new methods, we and others in the field have expressed concern about the feasibility and viability of these changes in certain locations [1,2]. Laboratories in high burden TB areas that perform diagnosis based on conventional microscopy are now for example recommended to move towards innovative liquid culturing systems for case detection and molecular line probe assays for drug susceptibility testing. These new technologies are more sensitive and rapid, but the concern is that they are also more prone to laboratory cross-contamination potentially resulting in misdiagnosis. This is especially problematic when these tests are performed in laboratories with inadequate infrastructure and maintenance, or laboratories that lack the possibility of confirmatory tests. This is often the case in countries with a high TB burden. The consequences of a high contamination risk are numerous, but essentially results in bacterial culture results from true negative samples becoming positive. This issue forms the focus of our study. As the treatment of TB patients is long and complex with the potential for side effects, a false-positive MTB test is not trivial and places a significant burden on the TB suspect, the suspect's family and the health system in general [3,4].

Many studies have investigated the risks, causes and consequences of laboratory cross-contamination both in low- and high burden TB settings. However, the effect of the prevalence of positive MTB samples in the laboratory - which is strongly associated with the TB prevalence in the area - is generally not explicitly considered with respect to the absolute risk of cross-contamination of a true negative sample. The aim of this study is to model how cross-contamination risk, in laboratories with different performance, is influenced by the rate of samples positive for MTB processed in the laboratory for liquid culture.

## Methods

First, a mathematical model is used to calculate the risk of a negative sample being tested false-positive based on various assumptions. It is assumed that the false positive rate (FPR) and false negative rate (FNR) are constant within a laboratory and depend only on the laboratory's performance. The variable factor under investigation is the prevalence of positive MTB samples (PSP) in the tested batch of samples, which influences the chance that a cross-contamination event will result in sample contamination with mycobacteria.

To determine the variables at stake a diagnostic 2 × 2 table is used (Table 1).

**Table 1:** Diagnostic 2 × 2 Table

		Reality		
		+	-	
Liquid culture	+	TP	FP	p
	
	-	FN	TN	q

A standard diagnostic 2 × 2 table visualizes the results of performing a diagnostic liquid culture test on a batch of samples from MTB suspects. Since liquid culture is the gold standard, the results are hypothetically compared to reality. The letters indicate different sample categories; TP true positive, FP false positive, FN false negative, TN true negative. p is number of samples tested positive by liquid culture, q is number of samples tested negative by liquid culture.

This theoretical framework provides us with the following variables:

- False positive rate (number of false positive samples divided by total number of positive samples) is F = FP/p

- False negative rate (number of false negative samples divided by total number of negative sample) is G = FN/q

The risk of a negative sample being tested falsely positive (called R) is calculated by dividing the false-positive samples (the false-positive rate times the positive TB samples) by the sum of the false positive and true negative samples; in a formula this is:

The number of true negative samples can be derived from the negative samples minus the false-negative samples (the false-negative rate times the negative samples). The new formula then becomes:

This formula can be applied to a wide array of different laboratory settings, ranging from laboratories with a very low FPR, FNR and PSP to laboratories with a very high FPR, FNR and PSP. Regarding the variables required, it should be noted that data on FPR and PSP are readily available from various field studies. In contrast, the FNR can not easily be measured in practice, since the gold standard is liquid culture. Thus we are in fact modelling the performance of the gold standard test. If we assume an increased FNR (G) for liquid culture, the effect will be an increase of R in the formula. Here we prefer to utilize the most conservative model minimizing R. Therefore, we assume that the FNR is zero (G = 0), creating the following simplified formula:

In reality of course there will be a small portion of false-negatives, meaning that we slightly underestimate R with our formula.

## Results

We used published study data from real-life laboratory settings to explore the implications of this formula. A study on culture cross-contamination rates in Dutch laboratories provides data representing situations with a low prevalence of TB and low levels of cross contamination[5]. In that study, all culture results of the TB national reference laboratory (NRL) were investigated over a seven-year period (1993 until 2000). The Dutch laboratories sent on average 1570 culture samples per year to the NRL, of which 21 were tested culture-positive by the NRL, leading to a mean PSP of 21/1570 = 0.0134 = 1.34%. Since the formula reflects rates, the number of positive and negative samples (p and q) can be replaced by the rate of positive and negative samples compared to the total number of samples in a batch (PSP and 1-PSP). The NRL also typed all positive TB cultures using DNA fingerprinting. It was estimated, that the FPR in this time period was 2.4% on average, ranging from 3.9% at the start to 1.1% at the end of the investigation. It should be noted, that the very low FPR of 1.1% (F = 0.011) was reached after cross-contamination investigations had been performed in the Dutch laboratories and advice had been given about how to minimize the FPR over a number of years. We assume this extremely low FPR to represent the maximum practically achievable specificity of the test under ideal conditions.

**Figure 1:**
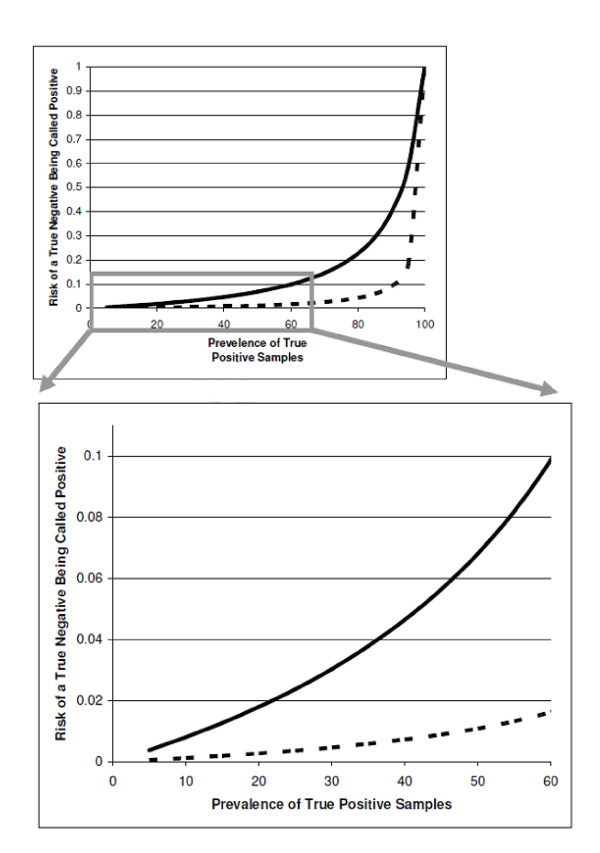
**Calculated risk of a negative patient sample being tested false positive due to cross contamination (Y axis) for different prevalence rates of positive MTB samples (X axis) showing the effect of the different laboratory cross contamination rates - 1.1% (dashed line) and 7.3% (solid line)**. The upper graph shows the exponential effect of the prevalence of true positives on contamination risk and the low graph shows an enlarged view of the clinically relevant area of the curve from the upper graph.

Using the above-mentioned data from the Dutch study, the risk of a negative sample becoming falsely positive can be calculated as followed:

This is the risk of a negative sample being cross-contaminated with a positive sample for a given FPR and PSP (1.1% and 1.34%, respectively). When the FPR in the laboratory is kept constant, but the prevalence of positive MTB samples increases, the relation between positive sample prevalence and cross-contamination risk can be visualized as shown in figure 1 (dashed line). It can be seen that the risk rises exponentially when the positive sample prevalence rises (upper graph).

In another published study, a South-African laboratory reported data representing a laboratory with a very high FPR and PSP[6]. In that study an average of 1186 culture samples per year were tested in the laboratory of which 55% were culture positive (PSP = 0.55, p = 1186 * 0.55 = 652). The FPR was reported to be 7.3% at the beginning of the study and after implementation of specific interventions it decreased to 2.1%. In order to model a broad range of realistic situation in this case we use the initial high FPR (F = 0.073) reported in the formula, the risk of contamination of a true negative culture can be calculated as follows:

With a constant FPR and increasing or decreasing the prevalence of positive TB samples in the South African laboratory, the relating contamination risks are depicted in figure 1 (solid line). Again, an exponential relation is seen between PSP and contamination risk.

Considering that a PSP of up to 55% does occur in high burden labs such as the South African laboratory, the part of the graph that is considered to represent a realistic situation is enlarged in figure 1 (lower graph). It is clear that when the FPR in a laboratory is kept constant, the risk of a sample getting cross-contaminated rises exponentially when the positive sample rate in the tested batch rises. Critically with a slightly raised FPR and high PSP this effect can quickly become a serious issue.

## Discussion

When applying diagnostic tests developed for use in situations with a very different pre-test probability of disease, it is informative to model the likely implications on specific aspects of test performance in the new situation. In this report we model the likely performance of *M. tuberculosis *automated liquid culture systems in high-burden countries.

Our calculations of the expected performance of liquid culture in different settings show that a laboratory with optimized procedures that achieves a low FPR (e.g. 1.1% - as for the Dutch laboratories) constitutes a low risk for a MTB negative TB suspect sample becoming contaminated, even when a high proportion of true positives are tested. When a contamination risk of 2.5% is taken as cut-off value (meaning that a higher contamination risk for a true negative sample is unacceptable, equivalent to a greater than 1 in 40 chance of a negative sample being falsely called positive), the PSP in a laboratory with a FPR of 1.1% due to cross-contamination needs to increase to over 60% in order for the contamination risk to rise above the cut-off value. Fortunately, a PSP of over 60% is unlikely in most countries. However, if the FPR is 7.3% - as for the South African laboratory when liquid culture was initially introduced -, the 2.5% cut-off value for contamination risk of a negative sample is reached when the PSP gets to 26%. With a FPR of 2.1% the PSP must reach 55% before the contamination risk of a true negative sample reaches 2.5%.

Over the previous years detailed and careful work has accurately defined the primary concern with automated liquid culture systems for the detection of *M. tuberculosis *infection. Namely, as a consequence of the comparatively rapid growth achieved and their excellent sensitivity they are more sensitive to sample cross-contamination than previous methods. We focused on this issue in this report. Once this problem is recognized the impact can be assessed and minimized. This results in a marked increase in performance in the years following adoption of the methods as well as the need for sufficient throughput and investment to maintain laboratory skills. Of course even in the best laboratories some level of cross-contamination is inevitable and is this report we aimed to be quite conservative regarding the likely cross contamination rates. In industrialized countries with functioning quality systems and active reference laboratory support this level appears to be around 1% of all positive samples [5].

The cross-contamination risk for true negative samples is not only dependent on a laboratories performance (mostly measured by FPR), but is also strongly influenced by the MTB PSP in the laboratory. Unfortunately, as our calculations demonstrate, the implications for even relatively low levels of cross-contamination are much more serious in locations with high TB prevalence in suspects. These laboratories are ironically at present often the least equipped to fully implement all the required protocols. These precautions include: separating the processing of non-sterile specimens and specimen with a high risk of contamination (e.g. specimen of known positive cultures, proficiency-test samples or contaminated culture vials); using individual aliquots of buffer solution; uncapping only one tube at a time; leaving samples at rest for five minutes after centrifugation or mixing; decreasing the maximum number of samples processed in one batch; re-enforcing hygiene protocols and clean laboratory practice among staff; and raising awareness and cautiousness of the possibility of false-positive results in general [7-9]. In order to monitor the performance of TB culture based diagnostic programs it is also useful to have the facility to genetically type a proportion of isolated strains, a service which is also frequently unavailable were it is most needed [10].

It has to be noted that in this study only the risk of cross-contamination leading to false-positive case detection is discussed. Like in most studies, the research articles used here focus on calculating the false-positive culture rate, i.e. the proportion of positive samples that is false-positive for the presence of MTB. However, they do not take into account the possible risk of a positive MTB culture sample being cross-contaminated with another positive sample, or the risk of a positive sample being contaminated with a (multi-)drug resistant MTB sample. The first event could potentially alter the *Mycobacterium tuberculosis *family type and lead to errors in determining epidemiological patterns. The latter could lead to a misdiagnosis of drug susceptibility of a sample and consequently to a patient receiving an incorrect treatment regimen. Thus, laboratory cross-contamination can also lead to erroneous detection of (multi-)drug resistance outbreaks or other epidemiological outbreaks. Since this study does not measure the risks of these other cross-contamination events, nor the effect that MTB positive sample rate might have on these risks, it is suggested that future studies also focus on these factors.

## Conclusions

It can be concluded that even if future global laboratory interventions are able to optimize the culture procedures leading to similar low false-positive culture rates in all laboratories around the world, the mathematical model described in this study predicts that the risk of contamination of negative cultures will inevitably remain higher in areas with a higher TB prevalence. It is likely that alternative liquid culture systems will have similar characteristics. Therefore, it is of critical importance that clinicians and policy makers involved in TB diagnostics remain aware of the possibility of laboratory cross-contamination, and take into account the regional differences when interpreting results and potential of these highly sensitive culture methods.

## Abbreviations

DNA: deoxyribonucleic acid; FPR: false-positive culture rate; FNR: false-negative culture rate; MTB: *Mycobacterium tuberculosis*; NRL: national reference laboratory; PSP: positive sample prevalence; TB: tuberculosis.

## Competing interests

The authors declare that they have no competing interests.

## Authors' contributions

All authors read and approved the final version of the manuscript. SK, RA and PK designed the study. SK created the initial mathematical model and performed a literary search to extract relevant data. Additionally, SK made calculations, developed graphs and drafted main body of manuscript. RA helped with and approved the mathematical model, partly drafted the manuscript and read and finalized the research paper. PK reviewed the research paper.

## Pre-publication history

The pre-publication history for this paper can be accessed here:

http://www.biomedcentral.com/1471-2334/10/93/prepub
